# Pain in an Internal Medicine Ward: An Undervalued Reality?

**DOI:** 10.7759/cureus.17838

**Published:** 2021-09-09

**Authors:** Inês Egídio de Sousa, Maria Teresa Neves, Cristina Gouveia, Renato Guerreiro, Luciana Frade, Teresa Mesquita, Luis Campos

**Affiliations:** 1 Internal Medicine, Hospital São Francisco Xavier, Centro Hospitalar de Lisboa Ocidental, Lisbon, PRT; 2 Oncology, Hospital São Francisco Xavier, Centro Hospitalar de Lisboa Ocidental, Lisbon, PRT

**Keywords:** pain, pain management, hospitalized, practicing internal medicine, internal medicine

## Abstract

Introduction

Pain is prevalent in most pathologic situations that require healthcare and is very common in hospitalised patients. However, there is limited evidence about the prevalence and the actual management of pain in medical wards. The aim of this work was to evaluate and characterise pain management in an internal medicine ward.

Methods

Retrospective analysis of clinical data of patients consecutively discharged from the internal medicine ward of a central tertiary hospital over a period of five months in 2018.

Results

199 patients evaluated, the median age was 78 years and 63% were female. Of these, 14% had a previous diagnosis of chronic pain, 24% were on chronic pain medication, and in 29% medication was interrupted. Pain was noted in medical records of 118 patients, with moderate to severe intensity in 67%. Among those, 71% had pain occurrence registered in the medical notes but not characterised in duration in 61%. The most common attributed etiologies of pain were musculoskeletal (16%), visceral (9%), and headache (8%); no identifiable cause was specified in 57%. In the group of patients reporting pain, 63% received analgesics. Opioids were used in 35% and 47% of patients with moderate and severe pain, respectively. At discharge, 12 patients were still referred pain, 16 had pain listed as a diagnosis, 45 were medicated for pain, and eight were referred for pain consultation.

Conclusions

Despite being highly prevalent in the internal medicine ward, pain is still under-recognised, undervalued, and under-treated. Education of healthcare staff and adoption of treatment protocols is essential to improve care for these patients.

## Introduction

Pain is defined as an unpleasant sensory and emotional experience associated with actual or potential tissue damage. It is a subjective and personal experience influenced to varying degrees by biological, psychological, and social factors [1].

Pain that lasts days or weeks is described as acute pain [2]. Acute pain is a biological mechanism that protects the body and maintains homeostasis. Pain indicates the presence of toxic stimuli and/or tissue damage, promotes healing, signalling to the affected to rest the affected area and/or seek help [2]. When pain persists for a period longer than three months it is considered chronic [3]. Chronic pain is a distinct condition in which pain persists beyond the normal time of healing of the lesion that originated it and is no longer just a symptom of injury or disease. Chronic pain is a medical problem on its own that should be recognised as a disease, as stipulated by the EFIC (European Federation of IASP) since 2001 [2,4].

Pain is prevalent in the majority of pathologic situations that require healthcare and is very common in inpatient wards [5-8]. Hospitalised patients may suffer pain from many causes, including manifestations of acute disease, pain related to treatments or medical procedures, and uncontrolled chronic pain. Uncontrolled pain tends to chronicity with a profoundly negative impact on a patient’s physical and mental condition and quality of life [9]. It increases morbidity and affects the recovery process with extended hospitalisations and higher healthcare costs [7,9].

The Portuguese National Program for Pain Control reinforces that pain control should be seen as a priority and health professionals must adopt strategies for prevention, evaluation, and control of pain [10]. In Portugal, the prevalence of chronic pain in the adult population is estimated to be 36.7%, with an average duration of 10 years. Of this population that experiences chronic pain, 85% are either continuous or recurrent and 68% grade their pain from moderate to severe [11].

Despite the high impact of pain, there is scarce information on its prevalence and management in patients in medical wards, making it difficult to raise awareness. The purpose of our work was to evaluate pain management in an internal medicine ward, more specifically its correct assessment, treatment, and referral.

## Materials and methods

We performed a cohort study of all adults consecutively discharged from the internal medicine ward of a central tertiary hospital, in a period of five months in 2018. Participants were excluded if they were hospitalised for social reasons or unable to consent. Written informed consent was obtained from all subjects or their legally authorised representatives, prior to study initiation.

The study consisted of an observational, descriptive, retrospective analysis of prespecified clinical data. Data was collected from individual electronic medical records including medical and nursing records. The following data was obtained for each patient: age, gender, history of chronic pain or chronic pain medication, follow-up in specialised pain consultation, assessed pain in vital signs records, pain register on electronic medical records and its characterisation (duration and cause), analgesics used during hospitalisation, pain specialist support in the management during hospitalisation, pain register as a diagnosis on discharging sheet, analgesics at discharge, and referral to a pain specialist. Pain intensity is assessed by nurses using the self-reported numeric rating scale (NRS) to record and chart pain intensity. We extracted pain intensity for each patient (maximum pain intensity and pain intensity at discharge) and categorised it into four levels for this study, according to the WHO Analgesic Ladder: no pain (0), mild pain (1-3), moderate pain (4-7), and severe pain (8-10) [12]. All data obtained were entered into a Microsoft Excel (Microsoft Corp., Redmond, WA) database; all staff involved in data collection were trained in order to standardise the procedure of data collection. Excel was used for the analysis of clinical data.

## Results

There were 199 patients discharged from the internal medicine ward in this period. Population demographics and personal history related to pain are shown in Table 1. Of the patients under chronic pain medication, 18 (38%) were under more than one drug and in 14 patients (29%) pain medication was interrupted during the hospital stay. Pain was noted in the medical records of 118 patients (59% of the population studied) who experienced pain during hospitalisation. Population pain demographics and pain characteristics are shown in Table 2. 

**Table 1:** Population demographics and pain history.

Age (years)		
Median (min-max)	78	(21-98)
Gender		
Female, n (%)	125	(63)
Pain history		
Previous chronic pain diagnosis, n (%)	27	(14)
Previous chronic pain medication, n (%)	48	(24)
Previous chronic pain consultation, n (%)	8	(4)

**Table 2:** Population with pain demographics and pain characteristics.

Age (years)		
Median (min-max)	78	(30-96)
Gender		
Female, n (%)	82	(69)
Pain intensity		
Light, n (%)	39	(33)
Moderate, n (%)	61	(52)
Severe, n (%)	18	(15)
Pain duration		
Acute, n (%)	38	(32)
Chronic, n (%)	8	(7)
Not characterised, n (%)	72	(61)
Pain type		
Musculoskeletal, n (%)	19	(16)
Visceral, n (%)	11	(9)
Headache, n (%)	9	(8)
Oncologic, n (%)	6	(5)
Trauma, n (%)	4	(3)
Ischemic, n (%)	2	(2)
Not characterised, n (%)	67	(57)

**Figure 1:**
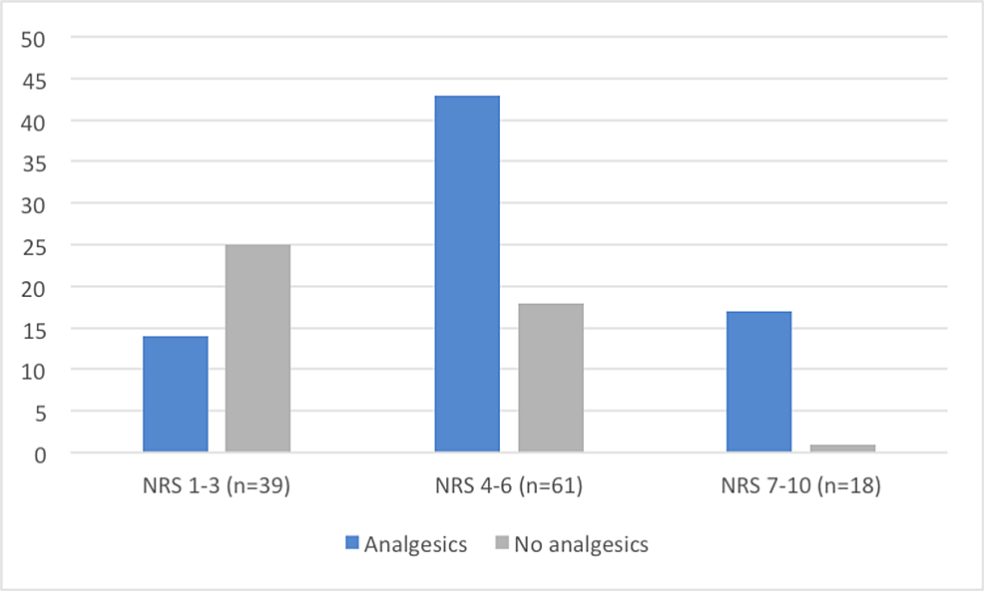
Administration of analgesics according to pain intensity. NRS - Numeric Rating Scale.

Among patients with pain, in 84 (71%) this was registered in the medical notes and 74 patients (63%) received analgesics during the hospital stay. We present analgesic administration according to pain intensity in Figure 1.

**Figure 2:**
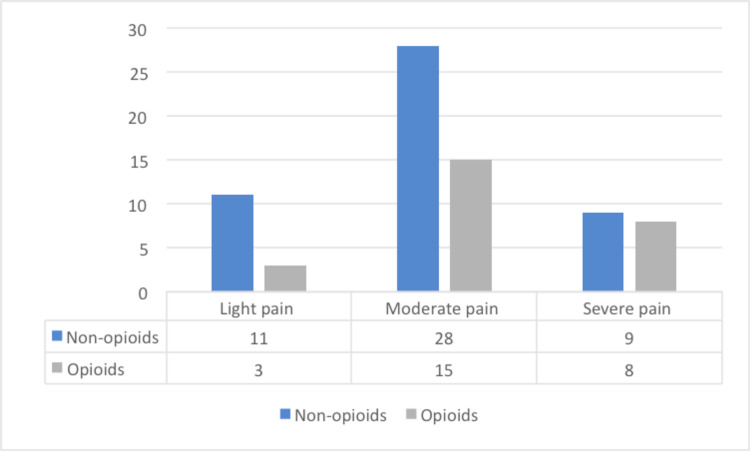
Opioid medication according to pain level.

The most widely used analgesics were paracetamol and metamizole/dipyrone for all types and degrees of pain (used in 58 patients and 28 patients respectively). Among patients who received analgesics for moderate pain, 35% received opioids. In patients treated for severe pain, 47% received opioids, and all others were medicated with non-opioid analgesics (Figure 2). In two patients, specialised consultation of pain was requested during hospitalisation. At discharge, 12 patients (10%) reported pain. From the population studied, 45 patients (23%) were discharged on pain medication and eight patients (4%) were forwarded to pain consultation. In 16 patients (8%), pain was registered as a diagnosis in the discharge letter.

## Discussion

This study is the first to evaluate pain prevalence, identification, characterisation, and management of a non-selected patient population in an internal medicine ward in a central hospital in Portugal. Little attention has been paid to pain in internal medicine wards, with publications mainly focused on the management of surgical or palliative patients. In our study, pain and its inadequate management were found to be a significant problem.

Of the patients studied, around one-fourth (24%) were previously under chronic pain medication, although only a few (14%) had pain diagnoses described on their personal history. This shows that despite the high prevalence of chronic pain in the general population in Portugal, this condition is still undervalued and underdiagnosed. The population studied was mainly women and elderly, known high-risk groups for chronic pain [8]. Pain management in the elderly and frail population is challenging due to age-related physiological changes causing pharmacokinetic and pharmacodynamic issues, the wide spectrum of comorbidities, and also the fact that the elderly tend to complain less even after experiencing pain [6,8,13]. Its inadequate recognition and treatment lead to a high probability of negative outcomes such as functional impairment, falls, slower rehabilitation, mood alterations, reduction of socialisation, deterioration of nutritional status, sleep disorders, and reduced quality of life [8].

Of the patients discharged in this period, more than half (59%) experienced pain, consistent with the high prevalence reported in previous studies in inpatient wards and reflecting the significant problem for hospitalised adults and their healthcare team [6,7,14,15].

The first step to improving pain control is to recognise and characterise the problem. In an effort to reduce the burden of underassessment and inadequate treatment of pain, in 1996, the American Pain Society (APS) instituted pain as the 'fifth vital sign' to make pain measurement as important for patient wellbeing as the existing four vital signs [16]. The campaign was widely supported and was later adopted in Portugal in 2003 [5]. We found that despite the records of pain as a vital sign, in many cases (29%) it was not valued and documented in the medical records. Also, in most patients who suffered from pain, it was not characterised both in duration (61%) or cause (57%). At the time of discharge, pain was considered in the diagnosis list of only 8% of the patients. These findings reflect how pain is still under-recognised and undervalued.

Pain relief is a human right and health professionals have the ethical and professional duty to adopt strategies to control pain effectively [4]. In this study, we found a significant number of patients (37%) who experienced pain and did not have analgesic prescriptions. We also find it remarkable that the proportion of patients with high pain levels was significant with most of the patients reporting moderate or severe pain (67%). Some patients (10%) still reported pain at the time of discharge. These findings reflect undertreatment and low control of pain in this population.

About the choice of the administered analgesics, we could verify insufficient adherence to the WHO recommendations on the use of analgesics, as only 35% of the patients with moderate pain and 47% with severe pain received opioids. These data reflect that despite recommendations on the use of analgesics according to the analgesic ladder, concerns about the use of opioids seem to persist [12]. Educational interventions are required to improve the knowledge and attitudes of health professionals handling patients in pain and specific education of health professionals in Portugal is still insufficient [4]. The use of scales and pain protocols are useful and easy to handle and should be used more widely in internal medicine wards.

Pain management can be very complex and its multidimensionality often requires a multi-professional and interdisciplinary therapeutic approach and it is within this scope that the specialised departments dedicated to pain medicine should be involved [17]. In the population studied, consultation by pain specialists was requested for two patients during hospitalisation and eight patients (4%) were referred to pain consultation follow-up at discharge.

Limitations of this study include its retrospective design and thus all limitations related to retrospective studies. The single-centre study is also a limitation. Collection of clinical data from medical records, with data not specifically addressed to evaluate pain features, therapy, and effectiveness in treatment, limits the accuracy of the information and, thus, limiting the interpretation of the results.

## Conclusions

We have shown that pain is highly prevalent in the population admitted to an internal medicine ward where the population is typically elderly and frail. Despite its relevance, pain is still under-recognised, undervalued and undertreated.

Pain relief should be a priority for health professionals, contributing to patients’ wellbeing, morbidity reduction, and humanisation of healthcare. Hopefully, this study has achieved its aims by raising awareness and sensitisation to the problem of pain and highlighting the need to improve its management in the clinical setting. Education of the healthcare team is essential to correctly assess, report, characterise, and effectively treat pain in order to improve care for these patients. Protocols for pain treatment and referral can also contribute to improving the quality of care and should be used more widely in internal medicine wards.
